# The novel treatment of children with viral warts using microwave technology

**DOI:** 10.1002/ski2.291

**Published:** 2023-09-18

**Authors:** Katie Solomon, Vincent Yip

**Affiliations:** ^1^ Department of Dermatology Alder Hey Children's NHS Foundation Trust Liverpool UK

## Abstract

Microwave therapy is emerging as an effective, well‐tolerated and safe treatment modality for cutaneous warts but there is limited data in paediatric patients. In our cohort of 35 paediatric patients with recalcitrant warts, 68.6% (24/35) demonstrated complete resolution after an average of three microwave treatment sessions. There were no reports of ulceration or blistering but 22.9% (8/35) of patients reported pain that required discontinuation of microwave therapy. This study provides evidence that microwave therapy can be used for the treatment of warts in paediatric patients.

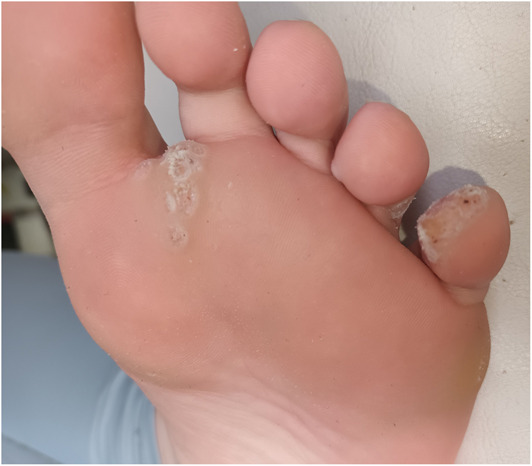

Dear Editor,

Cutaneous warts, caused by infection with human papilloma virus (HPV), are prevalent among school‐age children, with an estimated prevalence of up to 44%.^1^ These warts can cause pain, impair mobility, and result in psychological distress. Existing treatments for warts are suboptimal and encompass destructive treatments, virucidal agents, anti‐proliferative agents, and immunological therapies.^2^ In this context, microwave technology has emerged as a potential treatment option. Microwaves are part of the electromagnetic spectrum and can induce rapid heating to sub‐ablative temperatures (<50°C) to modulate immune responses against HPV infected cells.^3^ The Swift® microwave unit (Emblation Ltd.) is a medical device licenced in the UK, EU, and cleared in the US, which allows the delivery of microwave energy through the application of a probe.^4^ A previous study using microwaves has demonstrated efficacy for plantar warts in adult patients but there is limited data on its benefit and tolerability in a paediatric cohort.^5^

We reviewed the case notes for paediatric patients with warts who had completed a course of microwave treatment at a tertiary dermatology unit. Hyperthermia was induced at each treatment session using Swift through repeated applications lasting 1–2 s each, which supplied energy at a frequency of 8 GHz and an average power of 7W (SD: 2, range 2–10). Each wart received an average of four microwave applications per session (SD: 1, range 2–8). After the primary session, a follow‐up was arranged at approximately 4‐weekly intervals to assess clinical progression and to determine if further microwave treatments were necessary. Response to treatment was defined as binary: ‘resolved’ or ‘not resolved’. Resolution was defined as ‘clearance of the wart with a return of dermatoglyphics across the skin’. Tolerability was determined by asking patients if they were happy to continue with treatment. Patients who did not attend a follow up appointment were contacted by telephone to determine the outcome and tolerability.

The clinical characteristics and outcomes for patients are outlined in Table 1. There was a full clearance rate of 68.6% (24/35) among paediatric patients with a mixture of common and plantar warts (Figure 1). Patients who responded required an average of 3 (SD: 2.4, range 1–8) microwave treatment sessions for resolution. The average age of each wart was 29 months (range 6–84) and nearly all patients (94%) had already failed alternative treatments prior to microwave therapy emphasising the recalcitrant nature of the cohort. Microwave therapy was not tolerated in 22.9% (8/35) of patients due to pain.

**TABLE 1:** Characteristics and outcomes of patients treated with microwave therapy for warts.

Characteristics	Resolved (*n* = 24)	Not resolved (*n* = 11)
Sex (*n*)		
Male	13 (54%)	4 (36%)
Female	11 (46%)	7 (64%)
Age (years)		
Mean (range)	12 (6–17)	12 (6–19)
Duration of warts (months)		
Mean (median)	29 (24)	31 (36)
Previous treatments (*n*)		
Topicals	17	10
Cantharadin	11	5
Cryotherapy	2	1
Nil	2	0
Number of treatment areas		
Mean (range)	2 (1–3)	2 (1–4)
Number of treatment sessions		
Mean (range)	3 (1–8)	4 (1–6)
Tolerability		
Tolerated	20 (83%)	7 (64%)
Not tolerated	4 (17%)	4 (36%)

**FIGURE 1:**
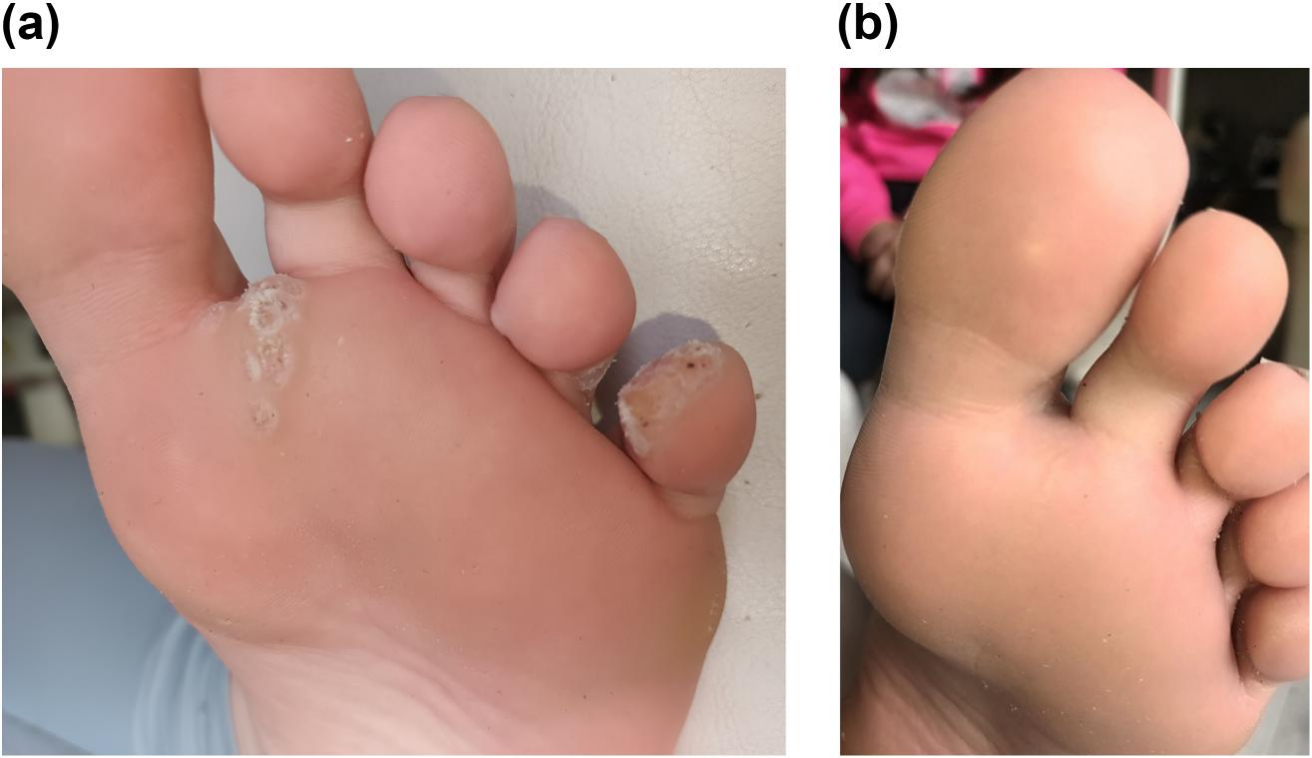
(a) Response of recalcitrant warts to microwave therapy; clinical image of plantar wart pre‐microwave treatment; and after four treatments (b).

This case series has demonstrated that microwave therapy can be used safely and effectively as a treatment modality for viral warts in children. Based on clinical experience, plantar warts are more resistant to therapy.^2^ In our cohort, 20 patients presented with plantar warts and clearance was achieved in 70% (14/20) of cases. This compares favourably to traditional treatments such as salicylic acid (31%) and cryotherapy (34%)^6^ and is consistent with previous rates of clearance using microwave therapy in adults.^5^ A study using cantharadin 1%, podophyllin 20% and salicylic acid 30% solution reported an 86.5% (45/52) clearance rate for all warts in children but was associated with adverse events such as blistering in 41.2% of patients.^7^ There were no reports of ulceration or blistering in our cohort although microwave therapy was associated with an acute short‐lived pain during the treatment that led to discontinuation in some patients. Adjusting the energy settings and duration of the device can improve tolerability.

Advantages of microwave therapy include short treatment time as each application takes approximately 2 s and most patients required only four applications each session. Patients do not require pre‐treatment preparation (e.g., local anaesthetic) and no special dressings or precautions are required post‐treatment allowing patients to continue with their regular activities. No episodes of scarring or pigmentary changes were reported in our study or previous studies meaning that microwave therapy may be particularly suited for warts in sensitive sites and skin of colour.^5^ In contrast to destructive modalities such as ablative lasers or cryosurgery, microwave therapy does not generate surgical plume eliminating the requirement for air extraction systems and minimising risks to operators from airborne viral particles. Microwave radiation is linear and non‐ionising thereby minimising injury to surrounding tissues and with no risk of DNA damage.^5^ Microwave hyperthermia of HPV‐infected cervical cells has been shown in vitro to inhibit cell proliferation and promote natural apoptosis of infected cells providing evidence for the mechanism of action in the treatment of viral warts.^8^ Limitations of our study include retrospective study design and lack of head‐to‐head comparisons. This study is among the first to report successful utility of microwave therapy for recalcitrant warts in a paediatric population who had failed other treatment modalities. Further randomised studies with larger sample sizes are warranted to confirm our findings.

## CONFLICT OF INTEREST STATEMENT

VY has received honoraria from Abbvie and UCB.

## AUTHOR CONTRIBUTIONS

**Katie Solomon**: Data curation (equal); formal analysis (equal); investigation (equal); methodology (equal); project administration (equal); writing—original draft (supporting); writing—review & editing (equal). **Vincent Yip**: Conceptualization (equal); data curation (equal); formal analysis (equal); funding acquisition (equal); investigation (equal); methodology (equal); project administration (equal); supervision (lead); writing—original draft (lead); writing—review & editing (equal).

## FUNDING INFORMATION

This work was supported by Emblation Ltd (UK).

## ETHICS STATEMENT

Not applicable.

## Data Availability

The data that support the findings of this study are available from the corresponding author upon reasonable request.
